# Trace elements in farmland soils and crops, and probabilistic health risk assessment in areas influenced by mining activity in Ecuador

**DOI:** 10.1007/s10653-023-01514-x

**Published:** 2023-03-01

**Authors:** Paola Romero-Crespo, Samantha Jiménez-Oyola, Bryan Salgado-Almeida, Johanna Zambrano-Anchundia, Cindy Goyburo-Chávez, Ana González-Valoys, Pablo Higueras

**Affiliations:** 1 Escuela Superior Politécnica del Litoral, ESPOL, Facultad de Ingeniería en Ciencias de La Tierra, Campus Gustavo Galindo km 30.5 vía Perimetral, P.O. Box 09-01-5863, Guayaquil, Ecuador; 2 Centro Experimental de Ingeniería, Universidad Tecnológica de Panamá, Vía Tocumen, P.O. Box 0819-07289, Panama City, Panama; 3 SNI-SENACYT Sistema Nacional de Investigación-Secretaria Nacional de Ciencia, Tecnología e Innovación, Clayton, Ciudad del Saber Edif.205, P.O. Box 0816-02852, Panama City, Panama; 4 Instituto de Geología Aplicada, Universidad de Castilla-La Mancha, EIMI Almadén. Almadén, 13400 Ciudad Real, Spain

**Keywords:** Heavy metal pollution, Agricultural soils, Toxicity, Food chain, Polluted crops

## Abstract

Consumption of food grown in contaminated soils may be a significant human exposure pathway to pollutants, including toxic elements. This study aimed to investigate the pollution level of trace elements in farmland soil and crops collected in orchards from Ponce Enriquez, one of the Ecuador’s most important gold mining areas. The concentration of arsenic (As), cadmium (Cd), chrome (Cr), copper (Cu), nickel (Ni), lead (Pb), and zinc (Zn) was analyzed in soil and crop samples (celery, chives, corn, herbs, lettuce, turnips, green beans, cassava, and carrots). In addition, a probabilistic human health risk assessment, in terms of hazard quotients (HQ) and cancer risk (CR), was conducted to assess the potential risk related to local crop ingestion. The contents of As, Cr, Cu, and Ni in soils exceeded the Ecuadorian quality guidelines for agricultural soils*.* The trace elements concentration in local crops was higher than the maximum permissible levels set by the Food and Agriculture Organization of the United Nations (FAO). The HQ and CR of local crop ingestion were several orders higher than the safe exposure threshold, mainly for lettuce, chives, and turnips. Our results revealed that inhabitants of the study area are exposed to developing carcinogenic and non-carcinogenic effects due to long-term food consumption with high trace elements. This study sheds light on the need to assess further the quality of agricultural soils and crops grown in mining areas with signs of contamination to guarantee consumer food safety.

**Supplementary Information:**

The online version contains supplementary material available at 10.1007/s10653-023-01514-x.

## Introduction

Food safety is a primary public health concern since food is the main source of human nutrition*.* However, consuming food grown in contaminated soils may be a major human exposure pathway to pollutants, including trace elements (Haque et al., 2021). As a result, trace elements pollution in the food system is a growing concern worldwide (Wang et al., 2019a, 2019b, 2019c). Furthermore, the intake of trace elements through the food chain is considered the main route of toxic metal pollution for humans and animals, leading to various diseases, including cancer, gastrointestinal disorders, and neurological problems (Kumar et al., 2022)*.*

Agricultural products can absorb contaminants from the soil and have high concentrations of trace elements (Haque et al., 2021). Food crops, particularly vegetables, are susceptible to soil pollution with trace elements. Leafy vegetables have been reported to be more likely to absorb, transport, and accumulate trace elements in their edible parts (Atikpo et al., 2021).

Trace elements can appear in agricultural soils naturally (i.e., atmospheric deposition, weathering, parent rock, or erosion) as well as through anthropogenic activities developed in the vicinity of these soils and by the same agricultural practices that incorporate various contaminants into the soil (Kumar et al., 2022). Among anthropogenic activities, mining is considered an important source of trace elements pollution since, when not carried out properly, it can release potentially toxic elements into the surrounding environment (González-Valoys et al., 2021; Jiménez-Oyola et al., 2021a, 2021b; Wang et al., 2019a, 2019b, 2019c; Xu et al., 2013).

Several studies have reported the presence of trace elements on farmland and foods grown near mining areas (Zhou et al., 2023). In countries like China, severe contamination of crops with trace elements in the neighborhood of mining areas has been reported (Wang et al., 2001; Zhang et al., 2015). Pollutants such as As, Cd, Cu, Hg, Pb, and Zn are transported by precipitation or flooding from contaminated mining sites to nearby farmlands and streams, causing environmental and human health risks (Li et al., 2014; Wang et al., 2019a, 2019b, 2019c). For example, in the city of Tongling, China, high concentrations of Cd, Cr, and Pb were detected in corn (*Zea mays*) grains, rice (*Oryza sativa*) grains, and vegetables near mining areas, exceeding China’s food safety limits (Wang et al., 2019a, 2019b, 2019c). Similarly, in crops around the Dabaoshan mine, the concentration of trace elements in rice soils exceeds the maximum concentrations allowed by Chinese legislation (Zhuang et al., 2009a, 2009b). In addition, the health risk assessment derived from the consumption of vegetables and rice by local inhabitants shows risk levels higher than the permissible limits of the Food and Agriculture Organization of the United Nations (FAO) (Zhuang et al., 2009a, 2009b). In other mining regions of China, such as the Shaoguan, Shandong, and Shaanxi Provinces, concentrations of trace elements were higher than the reference levels in agricultural soils (Li et al., 2018a, 2018b; Sun et al., 2018; Zhu et al., 2018). For example, in Nigeria, in the mining region of Enyigba, high concentrations of Cd, Cr, Pb, and Zn were found in tubers, vegetables, and fruits such as lemon (*Citrus limon*) and pumpkin (*Cucurbita pepo*) (Obiora et al., 2016). In this sense, there is ample evidence that crop areas near mining areas are vulnerable to contamination; food grown in these areas may pose a potential risk to consumers.

Ponce Enriquez is one of the most important gold mining fields in Ecuador. In addition to mining, this area has significantly developed its agricultural activity (MAE-PRAS, 2015). However, the previous studies in this place have shown the high content of trace elements in different environmental matrices such as water, soil, and sediment (Carling et al., 2013; Escobar-Segovia et al., 2021; Jiménez-Oyola et al., 2021a, 2021b; Tarras-Wahlberg, 2002). Furthermore, considering that rivers are used for irrigation of agricultural lands, their potential high content of trace elements could enhance non-essential toxic elements in the soils where plants are to be grown (Kumar et al., 2022; Yu et al., 2022).

Food production in contaminated lands can pose a potential risk to consumers since toxic contaminants such as As, Cd, and Pb, among others, can enter the human body through the food chain (Khan et al., 2015). In Ecuador, several studies have evaluated soil quality in agricultural areas. For example, Chavez et al. (2015) and Argüello et al. (2019) reported an accumulation of Cd in the soils of cocoa (*Theobroma cacao*) production in southern Ecuador. In addition, Barraza et al. (2018) identified high concentrations of trace elements in farmland in an oil extraction area of the Ecuadorian Amazon, and Dinter et al. (2021) studied the trace elements content in agricultural soils of the Galápagos Islands. However, no previous studies have evaluated the quality of agricultural soils and the content of trace elements in crops from mining areas with signs of pollution in Ecuador. For this reason, this study aims to: (a) assess the quality of the cultivation soil in agricultural orchards located in the Ponce Enriquez gold mining field, Azuay Province, Ecuador; (b) evaluate the presence of trace elements in locally grown foods; and (c) calculate the health risk to consumers of locally grown products.

## Materials and methods

### Study area

**Fig. 1:**
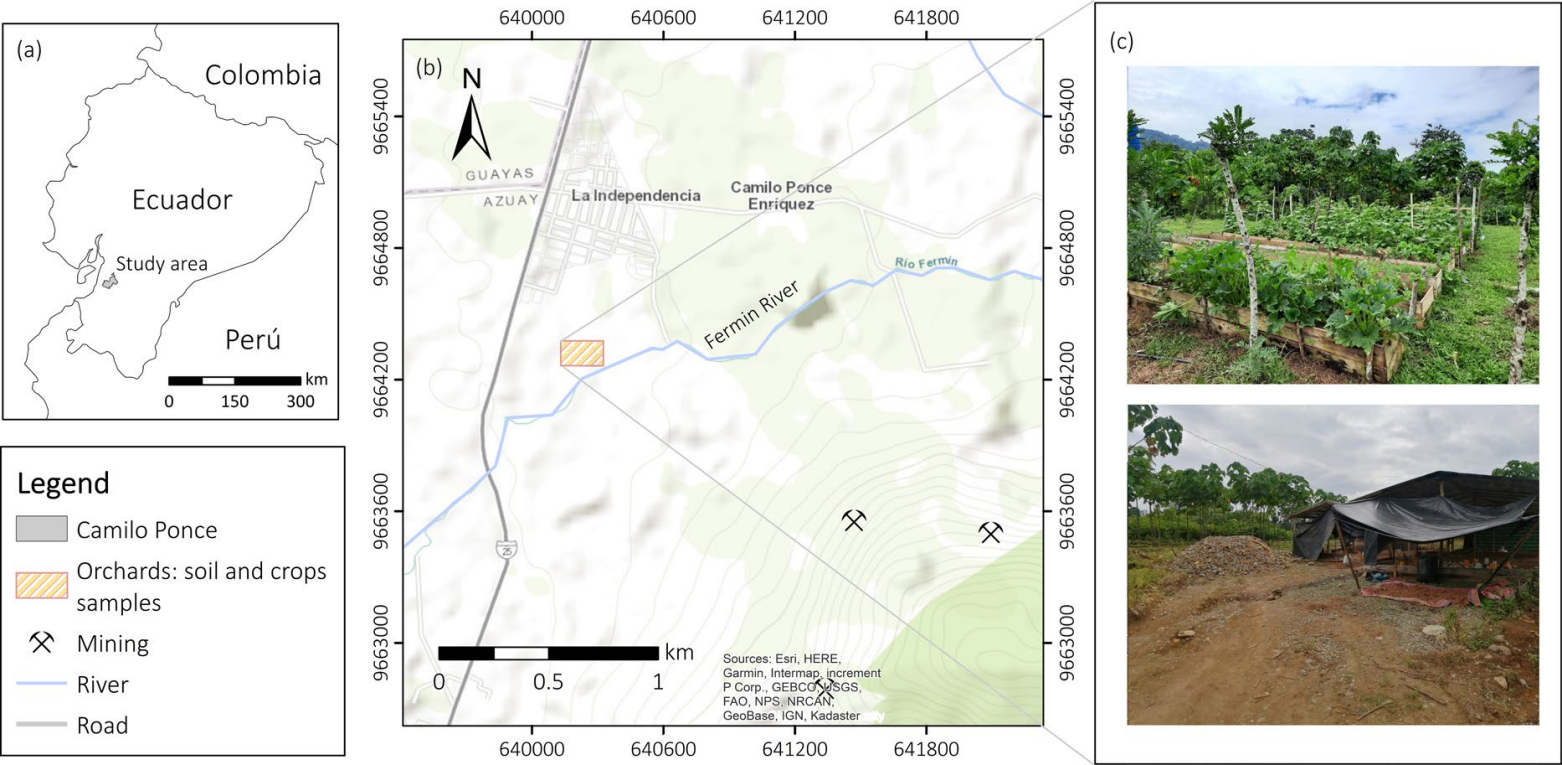
**a** Study area, **b** location of the study orchards in the mining area of Ponce Enriquez, and **c** orchards

The study area is in the Canton of Camilo Ponce Enriquez, located in the Azuay Province, southern Ecuador (Fig. 1a). It is a semi-humid area with an average annual rainfall of 1567 mm and a mean annual temperature of 24.8 °C (INAMHI, 2022)*.* The population is 21,998 inhabitants (INEC, 2010). Ponce Enriquez is one of the Ecuador’s oldest gold mining areas, with massive mining operations since the 1980s (Appleton et al., 2001). A large part of the mining operations is developed in the Bella Rica sector, where deposits are rich in sulfide and arsenic with common minerals such as pyrrhotite, arsenopyrite, chalcopyrite, galena, cuprite, chalcocite, covellite, and malachite (PRODEMINCA, 1998; Rivera-Parra et al., 2021). The extraction of gold is conducted by cyanidation and flotation. However, amalgamation with mercury is still used illicitly in the area. Other important socioeconomic activities in Ponce Enriquez are livestock (MAE-PRAS, 2015) and agriculture (coffee (*Coffea* sp.), cocoa, and plantain for exportation). This study focuses on local orchards managed by the Association of *Jancheras* (women mine-rock waste collectors) “*Unión y Progreso”* (Fig. 1b, c).

### Sampling collection and analyses 

The data correspond to local crop samples (*n* = 9) and farmland soil samples (*n* = 8) collected from agricultural orchards in the Ponce Enriquez gold mining area in June 2021. The seven target elements (As, Cd, Cr, Cu, Ni, Pb, and Zn) were analyzed in both soils and food samples. The soil analysis was carried out in the Analytical Chemistry Department of the Mexican Geological Service, Mexico, and the food analysis was carried out in the Plant Nutrition Laboratory of the Escuela Superior Politécnica del Litoral, Ecuador. The soil samples were taken from 5 to 20 cm depth with a shovel. Multiple samples were taken at each sampling point to obtain composite samples. The composite samples were air-dried, disaggregated, homogenized, quartered, grounded, and passed through a 2-mm sieve before their analyses. While the concentration of Cd, Ni, and Pb in soils was determined by Inductively Coupled Plasma Mass Spectrometer (ICP-MS) Agilent 7700x, the concentration of As, Cr, Cu, and Zn was determined using an optical emission spectrometer (Inductively Coupled Plasma Optical Emission Spectrometry) from Perkin–Elmer®, model AVIO 500 (Meier et al., 1994). Soil sample digestion was performed with four ultra-pure fatty acids (2.5HNO_3_-4HCl-HClO_4_-3HF), gradually heating on a hot plate (grill) from 70 to 160°C. The sample recovery stage is carried out with HCl and HNO_3_ to obtain the elements in an acid solution, either chlorides or nitrates, for subsequent quantification by ICP-OES or ICP-MS. During the analysis, reference materials NCS DC 73303 Rock and NCS DC 73507 Ore were used for quality control. The CCVs used are calibration curve verification standards, composed of the mixture of two high-purity liquid CRMs SP-928462712CHP and SP-928470213B, whose nominal value after preparation is 1 ppm. The percentage of recovery varied between 76 and 97%. The limit of detection (LoD) in mg kg^-1^ for the analyzed elements in soils was as follows: As = 4, Cd = 0.01, Cr = 3.33, Cu = 2.67, Ni = 0.01, Pb = 0.02, and Zn = 0.41. In addition, the pH was measured in deionized water (liquid/solid = 5/1) using a pH meter (Thermo Scientific Orion Star A215), and the organic matter (OM) content in soil samples was determined with a C/N analyzer (Vario MAX CN Macro Elemental Analyzer) (Chavez et al., 2015).

Crop samples were collected from the same sites where the soil samples were taken and included the main crops grown in the area: celery (*Apium graveolens*), chives (*Allium schoenoprasum*), corn, herbs (*Coriandrum sativum*), lettuce (*Lactuca sativa*), turnips (*Brassica rapa*), green beans (*Phaseolus vulgaris*), cassava (*Manihot esculenta*), and carrots (*Daucus carota sativus*). The food samples were washed with distilled water to eliminate soil and dirt particles. The edible part was cut into small pieces and was lyophilized, crushed, and homogenized. Then, 0.5 g of ground food samples were digested with 7 mL of concentrated nitric acid (HNO_3_) and 1 mL 30% H_2_O_2_ at 70 °C (90 mi) and 110 °C (up to 1 mL), and three replicates were digested per sample. The digesters were diluted to 25 mL with nano-pure distilled water and filtered through a 0.45-µm membrane filter prior to trace elements analysis (Chavez et al., 2015). The concentrations of trace elements were determined by triplicate using Inductively Coupled Plasma Optical Emission Spectrometry (ICP-OES) from Perkin–Elmer®, model OPTIMA 5300DV. The LoDs in mg kg^−1^ were as follows: As = 0.2, Cd = 0.004, Cr = 0.01, Cu = 0.04, Ni = 0.2, Pb = 0.1, and Zn = 0.1. Quality control was conducted by employing duplicates and certified reference material: NIST Standard Tomato Leaves 1573a.

### Statistical analysis

The data were statistically analyzed using the R-free software (R Core Team, 2020). Descriptive statistics were used to assess the trends of the data. The relationships among the metal(loid)s in both soil and food samples were evaluated through Spearman’s correlation coefficient. Furthermore, principal component analysis (PCA) was applied to investigate sources of contamination, anthropogenic activities, or geogenic sources (Yuswir et al., 2015).

### Exposure assessment and risk characterization

The main pathway of human exposure to trace elements is consuming contaminated food (Romero-Estévez et al., 2023). In the agricultural setting, other routes of exposure are accidental ingestion of soil and skin-to-soil contact during planting and harvesting activities; however, these routes do not significantly contribute to human health risks (Haque et al., 2021; Y. Zhou et al., 2018). In this study, the human health risk through ingestion of polluted local crops was quantified in terms of hazard quotients (HQ) and carcinogenic risk (CR), following the standard model of human health risk assessment recommended by the United States Environmental Protection Agency (USEPA). The assessment was performed for both adults and children. The average daily dose (ADD) was estimated using Eq. (1) proposed by the USEPA (2001, 2004).

1 $${\text{ADD}} = \frac{{C \times {\text{EF}} \times {\text{IRc}} \times {\text{ED}}}}{{{\text{AT}} \times {\text{BW}}}}$$

where C is the concentration of each trace element in food (mg kg^−1^, FW) on fresh weight; EF is the annual exposure frequency (days year^−1^); IR_c_ is the ingestion rate of local crops (kg day^−1^); ED is the exposure duration (years); AT is the averaging time (days); and BW is the body weight (kg).

The non-carcinogenic risk was calculated in terms of hazard quotients (HQ) using Eq. (2), and the Hazard Index (HI), which represents the cumulative non-carcinogenic risk, was estimated by the sum of the HQs. The carcinogenic risk (CR) was calculated according to Eq. (3), and the sum of the CRs estimated the cumulative carcinogenic risk or total cancer risk (TCR). The safe exposure threshold is exceeded for HI above one and TCR above 10^−5^ (USEPA, 2004).

2 $${\text{HQ}}_{{{\text{intake}}}} = \frac{{\text{ADD }}}{{{\text{RfD}}}}$$

3 $${\text{CR}}_{{{\text{intake}}}} = {\text{ADD}} \times {\text{SF}}$$

The values of reference dose (RfD) and slope factor (SF) were collected from the Risk Assessment Information System website (RAIS, 2022). RfD and SF were chosen assuming a conservative scenario. Therefore, the cancer risk was assessed only for As and Cr, which SF reported.

The probabilistic risk estimation was performed using a Monte Carlo simulation (MCS), a recognized method to determine the variabilities and uncertainties of human health risk-based evaluation (Saha et al., 2017). The probabilistic carcinogenic and non-carcinogenic risks were calculated using Oracle Crystal Ball. Ten thousand iterations were carried out for each simulation. The input variables annual exposure frequency (EF), exposure duration (ED), and body weight (BW) were introduced as a probability distribution function. On the other hand, trace elements concentration (*C*_i_), ingestion rate (IR_c_), average time (AT), reference dose (RfD), and slope factor (SF) were introduced in the risk model as a point estimate. Table 1 shows the distributions and point values used in the risk assessment through the ingestion of local crops for both adult and children receptors.

**Table 1:** Parameters and values used for the probabilistic assessment

Parameter	Units	Probabilistic approach		References
		Distribution	Values	
EF	day year^−1^	Triangular	345 (180–365)	(USEPA, 1996)
ED_*a*_	year	Lognormal	11.36 ± 13.72	Israeli and Nelson (1992)
ED_*c*_	year	Uniform	1–6	Spence and Walden (2001)
Bw_a_	kg	Normal	60.39 ± 8.09	Jiménez-Oyola et al. (2021a, 2021b)
Bw_*c*_	kg	Normal	15.6 ± 3.7	Anderson et al. (1985)
IR_*c*_	kg day^−1^	–	0.17	Islam et al. (2014)
AT_nc_	day	–	365 × ED	USEPA (2004)
AT_ca_	day	–	365 × 70	USEPA (2004)
RfD	mg kg^−1^ day^−1^	–	As (0.0003), Cd (0,0001), Cr (0.003), Cu (0.04), Ni (0.02), and Zn (0.3)	RAIS (2022)
SF	(mg kg^−1^ day ^−1^)^−1^	-	As (1.5) and Cr (0.5)	RAIS (2022)

EF, exposure frequency; ED_a_, exposure duration adult; ED_c_, exposure duration children; Bw_a_, body weight adults; Bw_c_, body weight children; IR_c_, ingestion rate; AT_nc_, averaging time non-carcinogen; AT_ca_, averaging time carcinogen; RfD, reference dose; and SF, slope factor

## Results

### Trace elements content in soil samples

**Fig. 2:**
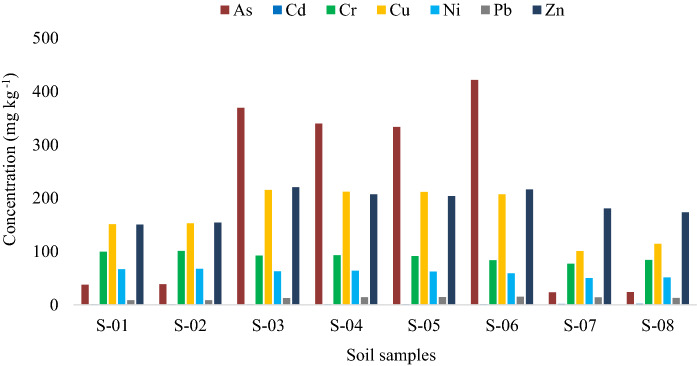
Trace elements content in soil samples from orchards

The physicochemical parameters measured in the agricultural soils of the study area are shown in Table S1 and Fig. 2. The pH in soils from Ponce Enriquez varied between 6.27 and 7.58, within the recommended limits for agricultural soils (6 < pH < 8) according to Ecuadorian regulations (TULSMA, 2015). The organic matter (OM) content ranged from 1 to 6% (Table S1), with five samples in the category of rich in OM (OM > 3%) and three samples with poor to acceptable OM content (1% < OM < 3%) (Ibarra et al., 2006).

The 50th percentile of the trace elements content in soils was in the decreasing order of Zn > As > Cu > Cr > Ni > Pb > Cd. The concentration of trace elements varied significantly between the samples in the following range: As (23.54–421.74 mg kg^−1^), Cd (1.06–1.94 mg kg^−1^), Cr (77.24–101.04 mg kg^−1^), Cu (100.91–215.59 mg kg^−1^), Ni (50.21–67.58 mg kg^−1^), Pb (8.69–15.16 mg kg^−1^), and Zn (150.52–220.06 mg kg^−1^). Considering the Ecuadorian quality guidelines (EQG) for agricultural soils, As, Cr, Cu, and Ni were above the recommended concentration (As = 12, Cr = 65, Cu = 63, and Ni = 50 mg kg^−1^) in all farmland samples. Zn was above the EQG (Zn = 200 mg kg^−1^) in 50% of the samples, but Cd and Pb were below the EQG (Cd = 2 and Pb = 60 mg kg^−1^) in all samples. Based on the results, trace elements pose a high potential ecological risk to the agricultural soil in the Ponce Enriquez mining areas. Similar findings were informed by Zhu et al. (2018) in agricultural soil in three mining areas in China.

The concentrations of Cd and Zn from Ponce Enriquez were in the range reported by Orellana Mendoza et al. (2021) in areas influenced by mining activity in the central region of Peru; yet, the content of Pb (72.11 ± 15.43 mg kg^−1^) was lower in our study. In contrast, the As concentration in the previously mentioned research (20.85 ± 5.79 mg kg^–1^) was several times lower than ours. Furthermore, the concentrations of Cd, Cr, Cu, and Pb measured in our study were found in residential areas (Jiménez-Oyola et al., 2021a, 2021b) from Ponce Enriquez, with values of concentration (p50) of Cd = 0.96 mg kg^−1^, Cr = 74.32 mg kg^−1^, Cu = 83.81 mg kg^−1^, and Pb = 11.12 mg kg^−1^. However, in the study above, low concentrations of As (p50 = 4.28 mg kg^−1^) and high contents of Ni (p50 = 176 mg kg^−1^) were also reported. Similar results were reported by Kumar et al. (2022) in agricultural soil samples from India, with contents of As (0.39–56.35 mg kg^−1^) lower than those detected in farmland soils from Ponce Enriquez. This research shows that the high content of trace elements in the agricultural soils from the Ponce Enriquez mining area can pose a risk for inhabitants who consume locally grown food. These results highlight the need to monitor the quality of agricultural soils and crops in potentially polluted mining areas.

### Trace elements content in food samples

**Fig. 3:**
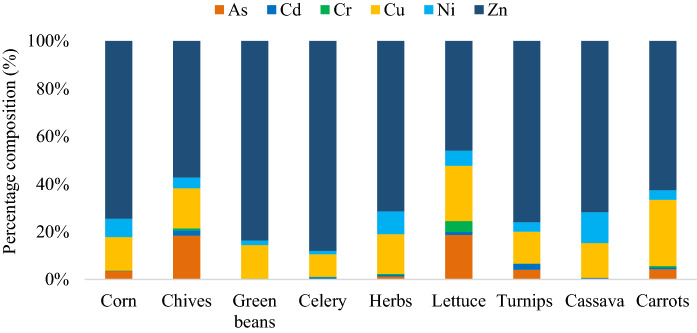
Percent composition of trace elements in local crop samples

The concentration of the trace elements in local crop samples (mg/kg, FW) on fresh weight is shown in Table 2. The trace elements concentration (p50) followed a decreasing order as Zn > Cu > Ni > As > Cr > Cd (Fig. 3). Pb was below the detection limit of 0.10 mg kg^−1^ in all samples. The results obtained in this study show significant variations in trace element concentration among food samples. These differences may be due to the variation in the absorption and accumulation capacities of the investigated species (Islam et al., 2014).

**Table 2:** Concentration of trace elements in food samples (in mg/kg) based on fresh weight

Crop	As	Cd	Cr	Cu	Ni	Pb	Zn
Corn	0.74	0.01	*	2.96	1.62	*	15.64
Chives	8.42	1.01	0.38	7.66	2.06	*	26.11
Green beans	*	*	*	5.52	0.72	*	32.71
Celery	*	0.72	0.17	7.89	1.21	*	74.01
Herbs	1.25	0.55	0.49	15.88	9.10	*	68.22
Lettuce	12.50	0.81	3.04	15.48	4.23	*	30.71
Turnips	2.55	1.64	*	8.40	2.55	*	47.90
Cassava	*	0.06	0.01	2.62	2.35	*	12.91
Carrots	1.94	0.33	0.30	12.62	1.84	*	28.39
MPL	0.10	0.05	2.30	40	10	0.10	20.00

^*^Below the limit of detection

MPL: maximum permissible levels set by the FAO/WHO (2021)

The results obtained in this study were compared with the maximum permissible levels (MPL) set by the FAO/WHO (2021). For As, 67% of the samples exceeded the limit of 0.10 mg kg^−1^, with the highest values in lettuce (12.50 mg kg^−1^) and chives (8.42 mg kg^−1^), which suggests that As might cause toxic effects through the consumption of these crops. Cd was above the recommended FAO/WHO safe limit in 78% of the samples; only corn and green beans reported values of Cd below the MPL. Cr exceeded the MPL of 2.30 mg kg^−1^ only in lettuce. Zn concentration was lower than the MPL of 20 mg kg^−1^ in corn and cassava samples. Cu and Ni were within the FAO/WHO guideline values in all food samples.

Considering the food safety standards, As and Cd were the pollutants of most significant concern since it was up to 120 times higher than the permissible level recommended by the FAO/WHO in the lettuce sample, which indicates that it is unsafe for human consumption. Turnips and chives were the food samples with the highest content of Cd, with values of 1.64 mg kg^−1^ and 1.01 mg kg^−1^, respectively, exceeding the recommended value (0.05 mg kg^−1^) by more than 20 times.

Several studies have reported the presence of potentially toxic elements on agricultural soils and foods grown near mining areas. For example, Hayford et al. (2009) reported a high concentration of trace elements in cassava and plantain samples collected from gold mining communities in Ghana. Liu et al. (2019) informed about Tl pollution in farmlands and vegetables in a pyrite mining area in China. Svoboda et al., (2006), and Zhuang et al., (2009a, 2009b), reported elevated trace elements in agricultural soils and crops from mining areas in the Czech Republic and China, respectively. Similar findings were informed in areas influenced by mining activity in the central region of Peru, where the mean concentration of Cd (0.32 ± 0.23 mg kg^−1^) and Pb (0.20 ± 0.12 mg kg^−1^) in *Lepidium meyenii Walpers* (maca) samples exceeded the values established by the FAO/WHO guideline (Orellana Mendoza et al., 2021). In contrast, Marrugo-Madrid et al. (2022) determined trace element contents below the threshold established in the regulations in fruits and tubers collected in the gold mining area of Colombia. Therefore, the degree of contamination will depend on the type of mining, geogenic conditions, and environmental factors that favor the release and mobility of pollutants.

### Spearman correlation and principal component analysis 

Spearman correlation analysis provides information regarding associations of trace elements in each medium. For soil samples, strong positive correlations (*r* ≥ 0.8) were found between As–Cu and Ni–Cr, and high positive correlations (*r* ≥ 0.7) between As–Zn and Cu–Zn. High negative correlations (*r* ≥ 0.8) were found between Cd–Cr and Cd–Ni (Table 3). For crop samples, there was a strong positive correlation (*r* ≥ 0.8) between Cr and Cu and moderate positive correlations (*r* ≥ 0.6) between As–Cd, As–Cr, As–Cu, and Cu–Zn. The statistical analysis did not include Pb in food samples because its concentration was below the LoD of 0.10 mg kg^−1^. The higher correlations among trace elements might indicate similar pollution levels and familiar sources of those elements in farmland soils.

**Table 3:** Correlation matrix of trace elements concentration in soils (mg/kg) and crops samples (mg/kg, FW)

Media		As	Cd	Cr	Cu	Ni	Pb
Soils	Cd	− 0.41					
	Cr	0.26	− 0.80				
	Cu	0.90*	− 0.51	0.46			
	Ni	0.48	− 0.88*	0.95*	0.58		
	Pb	0.33	0.48	− 0.61	0.18	− 0.46	
	Zn	0.76*	0.26	− 0.38	0.71	− 0.29	0.62
Crops	Cd	0.75*					
	Cr	0.73*	0.50				
	Cu	0.63	0.50	0.85*			
	Ni	0.52	0.53	0.47	0.58		
	Zn	0.08	0.33	0.41	0.62	0.05	–

^*^p-value < 0.05

For soil and food samples, two principal components (PC) were extracted with eigenvalues > 1 (Table 4). For soils, PC1 and PC2 explained 96.40% of the variance (PC1 = 49.80% and PC2 = 46.59%). Cu and Ni had significant loading on PC1, while Cr and Ni had significant loading on PC2. For food samples, 74.60% of the variance was explained by PC1 (48.46%) and PC2 (26.148%). As, Cr, and Cu had significant loading on PC1 and Zn and Ni in PC2. The PCA outputs suggest that Ni, Cu, Cr, and As are the primary contaminants of farmland soil because of anthropogenic sources, which is consistent with the previous studies by Jiménez-Oyola et al., (2021a, 2021b), who reported high contents of Cr, Cu, and Ni in soils from Ponce Enriquez.

**Table 4:** Principal component analysis (PCA) of trace elements concentration in soil (mg/kg) and crops samples (mg/kg, FW)

Parameter	Farmland soil		Local crops	
	PC1	PC2	PC1	PC2
As	0.317	− 0.443	0.425	− 0.511
Cd	− 0.499	− 0.096	0.334	0.124
Cr	0.408	0.336	0.459	− 0.411
Cu	0.450	− 0.293	0.530	0.142
Ni	0.495	0.205	0.391	0.326
Pb	− 0.122	− 0.528	–	–
Zn	0.149	− 0.524	0.247	0.653
Eigenvalues	3.480	3.260	2.907	1.568
% of variance	49.80	46.59	48.46	26.14
Cumulative %	49.80	96.40	48.46	76.40

### Human health risk

**Fig. 4:**
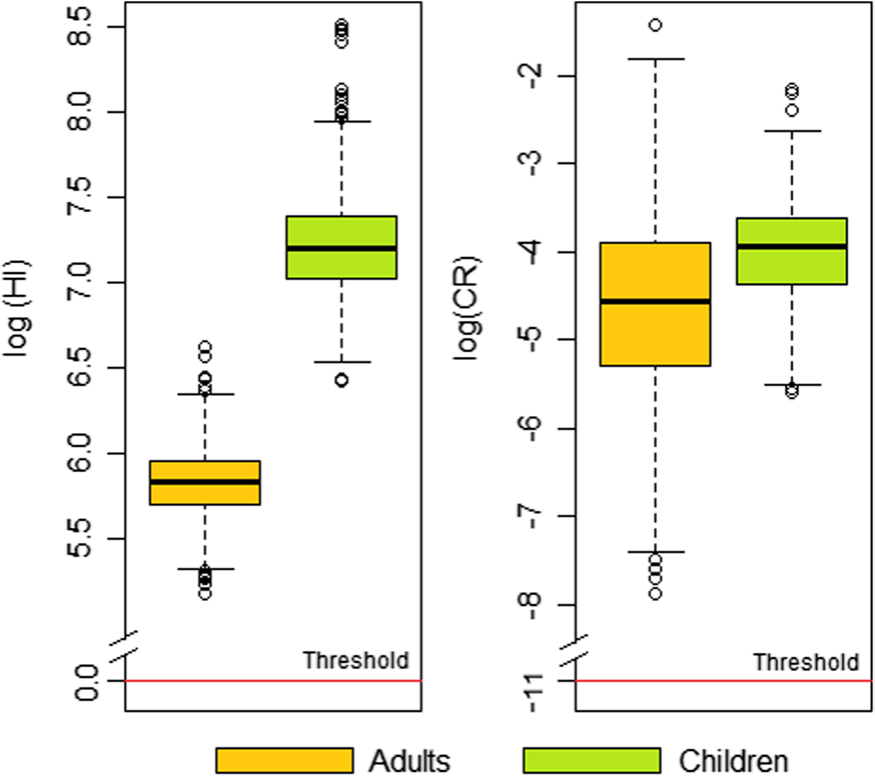
Probabilistic Hazard Index (HI) and total cancer risk (TCR) for adults and children through ingestion of local crops in the Ponce Enriquez mining area

The human health risk from exposure to trace elements through ingestion of polluted local crops was assessed for both local adults and children. Figure 4 shows the Hazard Index (HI) and the total carcinogenic (TCR) indexes. The USEPA above the safe exposure limit recommended the risk outcomes by probabilistic methods for both receptors for the 95th percentile for non-carcinogenic and carcinogenic risk (Tables S2 and S3, respectively).

**Fig. 5:**
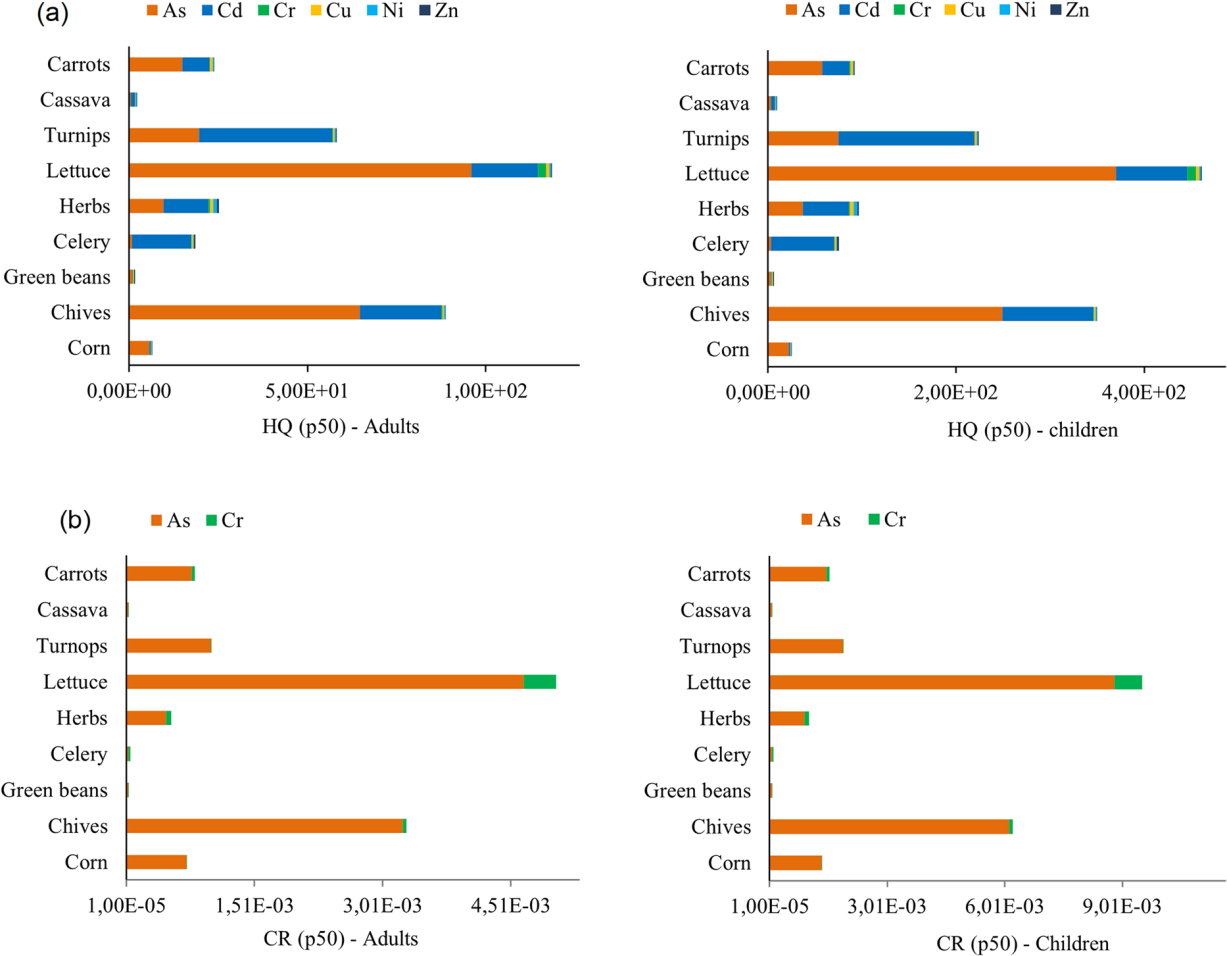
Non-carcinogenic risk (HQ) and carcinogenic (CR) by crop species for both adults and children

The HQ value exceeded the safe limit of one for adults and children in all the food samples. The HQ of each trace element through local crops consumption decreased in the order of As > Cd > Cu > Cr > Ni > Zn. In this line, As and Cd were the main contributors to the non-carcinogenic risk, with 95% of the Hazard Index (HI). Children were the most vulnerable receptor; the HQ for children was four times higher than the HQ for adults. The non-carcinogenic risk in children and adults increases in the following order: lettuce > chives > turnips > herbs > carrots > celery > corn > cassava > green beans (Fig. 5a). Regarding carcinogenic risk, the acceptable CR value is exceeded by As and Cr in all the food samples, being As the most significant contributor to the cancer risk, with 95% of the TCR. Lettuce and chives were the food samples of major concern (Fig. 5b), with CR values significantly higher than the safe exposure limit. The probabilistic risk assessment revealed that 100% of the consumers of local crops might be exposed to carcinogenic and non-carcinogenic health effects caused by the ingestion of food contaminated with trace elements. Similar findings were reported in mining areas of Panamá (González-Valoys et al., 2021), Peru (Orellana Mendoza et al., 2021), and Central China (Li et al., 2018a, 2018b), where the presence of potentially toxic elements in edible plants exceeded the safe exposure limit for non-carcinogenic and carcinogenic risk related to crop ingestion.

## Discussion

Food crops, mainly vegetables, are the primary source of human exposure to trace elements, contributing to about 90% of the total intake (Khan et al., 2015). Therefore, assessing the quality of food grown in contaminated areas is necessary to assure consumer food safety. In our study, local crops are severely polluted with trace elements. The concentration of As, Cd, Cr, and Zn was above the maximum permissible levels set by the FAO/WHO (2021), mainly in chives, turnips, and lettuce, the latter known as a hyperaccumulator of trace elements (Khan et al., 2015; Li et al., 2006).

High concentration of trace elements in agricultural soils has a negative impact on crops (Khan et al., 2015). In Ponce Enriquez, the agricultural soil samples showed extremely high concentrations of As (23.54–421.74 mg kg^−1^), exceeding up to 35 times the limit established in the Ecuadorian regulations (TULSMA, 2015). In many agricultural areas, contamination and loss of soil quality are often associated with poor quality irrigation water (Romero-Estévez et al., 2023). In Ponce Enriquez, the high concentration of trace elements in the rivers has been widely reported (Appleton et al., 2001; Carling et al., 2013; Escobar-Segovia et al., 2021; Jiménez-Oyola et al., 2021a, 2021b). Therefore, its use for irrigation may contribute to soil and food contamination in the area.

Food safety has been a primary public health concern in recent decades, as food crops may contain trace elements resulting from high levels of environmental pollution (Kumar et al., 2019). Some trace elements such as Cu, Cr, and Zn are essential for humans since they play an important role in different metabolic functions. However, other elements such as As, Cd, and Pb are non-essential and have high toxicity (Khan et al., 2015), showing carcinogenic effects (RAIS, 2022).

**Fig. 6:**
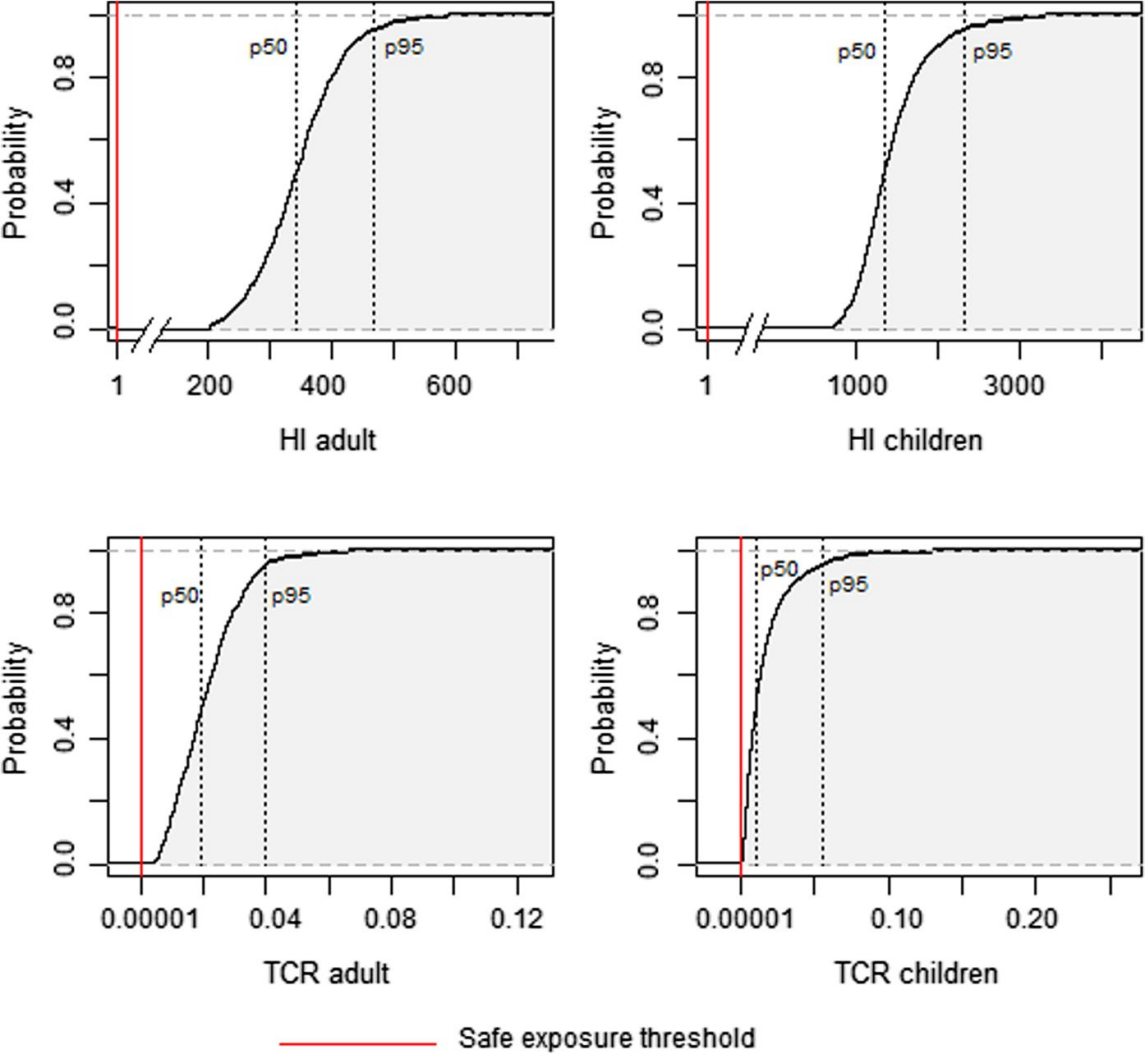
Cumulative probability distribution for Hazard Index (HI) and total cancer risk (TCR) for adults and children. The HI and TCR were above the safe exposure threshold for all percentiles, indicating that 100% of the receptors are exposed to non-carcinogenic and carcinogenic risk

The presence of trace elements in crops from the study area showed unacceptable levels of risk to consumers’ health. Lettuce was the crop with the most significant contribution to both non-carcinogenic and carcinogenic risk, which poses a concern since lettuce is a highly consumed vegetable in Ecuador (Romero-Estévez et al., 2020). The human health risk of crops consumption was several orders of magnitude higher than the safe exposure threshold, and the trace elements with the greatest contribution to the risk were As and Cd. Both As and Cd are considered human carcinogens (IARC, 1987), whose effects on human health have been widely reported in the literature. As exposure is associated with elevated cancer risks, i.e., bladder, blood systems, kidney, skin, lung, liver, and colon (Rashid et al., 2019), and Cd ingestion can cause diarrhea, stomach pain, fractures due to bone weakening, reproductive problems, damage to the central nervous system, damage to the immune system, and psychological problems (ATSDR, 2022; RAIS, 2022). In Ponce Enriquez, the probability of an individual developing cancer over a lifetime because of intake of trace elements, mainly As, was higher than acceptable levels (Fig. 6). In this line, policies strategic on reducing exposure to polluted food are needed to avoid the detrimental health effects of consumers.

In recent years, various strategies have been evaluated to reduce the bioavailability of toxic elements from the soil to the plant, including nanotechnological tools (Kumar et al., 2019). Several studies suggest that crop selection could be used to reduce the presence of trace elements in food grown in polluted soils, thus protecting consumers’ health. For example, Cao et al. (2022) found that tomatoes (*Solanum lycopersicum*), kidney beans, potatoes, and cabbage grown in contaminated soils show lower risks for consumers than other food species. Zeng et al. (2021) reported that fruits, root, and tuber vegetables had lower As accumulation than some leaf vegetables. Rodriguez-Iruretagoiena et al. (2015) concluded that tomato plants grown in soils with high concentrations of trace elements did not show high content of contaminants in their fruits. Therefore, selective planting of species with low levels of trace element accumulation should be implemented in the study area. This study highlights the importance of controlling the quality of agricultural soil and local crops in Ponce Enriquez. Public health and environmental supervision institutions must monitor the impact of anthropogenic activities in the area and adopt effective measures to reduce the risk to human health from exposure to contaminants through the food chain.

## Conclusions

This research is the first to determine the content of trace elements in soil and crops from agricultural orchards in the Ponce Enriquez gold mining area and to assess the human health risks through local crop ingestion. The findings of this study revealed that the concentrations of As, Cr, Cu, and Ni in farmland soils exceed the Ecuadorian quality guidelines for agricultural soils in all samples. On the other hand, the concentrations of As, Cd, and Zn in local crops were higher than the maximum permissible levels set by the Food and Agriculture Organization of the United Nations (FAO). The concentrations of trace elements in the food samples varied significantly as a function of plant species. Lettuce and chives are the crops of most significant concern. The Hazard Index (HI) and the total cancer risk (TCR) values from consuming local crops, mainly lettuce, chives, and turnips, were several orders of magnitude higher than the safe exposure threshold for adults and children. The probabilistic health risk revealed that 100% of consumers in the study area have a significant possibility of suffering carcinogenic and non-carcinogenic health effects caused by consuming crops with high trace elements. Therefore, this study recommends continuous monitoring of the quality of agricultural soils and crops grown in the Ponce Enriquez mining area. Based on the results obtained, it is recommended to prohibit the cultivation of plants that hyperaccumulate potentially toxic elements. It must be considered that even the inedible parts of these foods must be properly treated as waste to prevent these products from being consumed by animals and reaching humans through the food chain. Considering the high concentration of trace elements in agricultural soils, the planting of crops that do not accumulate trace elements in their edible parts is recommended to minimize the risk to the population. The risk outcomes of this study need to be further investigated; population-specific parameters, mainly the ingestion rate of local crops, should be determined locally to obtain more accurate risk outcomes. Overall, further studies are required to evaluate the availability of trace elements in soils and the soil–plant transfer processes. The use of fertilizers that can add potentially toxic elements to the soil should be avoided. In addition, agricultural soil protection and rehabilitation measures must be implemented to reduce the risk of transferring toxic elements to the food chain.

### Supplementary Information

Below is the link to the electronic supplementary material.

Supplementary file1 (PDF 109 KB)
