# Supplementary material for: Trace elements in farmland soils and crops, and probabilistic health risk assessment in areas influenced by mining activity in Ecuador
Source: Environ Geochem Health. 2023 Mar 1;45(7):4549–63. doi: 10.1007/s10653-023-01514-x (PMC10310628; doi:10.1007/s10653-023-01514-x)
Supplement: Supplementary file 1 — Supplementary file1 (PDF 109 KB) [file 10653_2023_1514_MOESM1_ESM.pdf]

Environmental Geochemistry and Health

Supplementary materials

Trace elements in farmland soils and crops, and probabilistic health risk assessment in areas influenced by mining activity in Ecuador.

Paola Romero-Crespo [1], Samantha Jiménez-Oyola [1*], Bryan Salgado-Almeida [1], Johanna Zambrano-Anchundia [1], Cindy Goyburo-Chávez [1], Ana González-Valoys [2,3], Pablo Higueras [4]

[1] Escuela Superior Politécnica del Litoral, ESPOL, Facultad de Ingeniería en Ciencias de la Tierra, Campus Gustavo Galindo km 30.5 vía Perimetral, P.O. Box 09-01-5863, Guayaquil, Ecuador; plromero@espol.edu.ec, 0000-0002-3751-2662 (P. Romero-Crespo); sjimenez@espol.edu.ec, 0000-0002-3538-6754 (S. Jiménez-Oyola); bryjosal@espol.edu.ec, 0000-0002-7188-8081 (B. Salgado-Almeida); jolizamb@espol.edu.ec, 0000-0002-3345-1890 (J. Zambrano-Anchundia); cgoyburo@espol.edu.ec (C.Goyburo-Chávez);

[2] Centro Experimental de Ingeniería, Universidad Tecnológica de Panamá, Vía Tocumen, Panama City 0819-07289, Panama; ana.gonzalez1@utp.ac.pa, 0000-0001-5963-2289 (A. González-Valoys)

[3] SNI-SENACYT Sistema Nacional de Investigación-Secretaria Nacional de Ciencia, Tecnología e Innovación, Clayton, Ciudad del Saber Edif.205, Panama City 0816-02852, Panama; ana.gonzalez1@utp.ac.pa, 0000-0001-5963-2289 (A. González-Valoys)

[4] Instituto de Geología Aplicada, Universidad de Castilla-La Mancha, EIMI Almadén. Almadén, 13400 Ciudad Real, España; pablo.higueras@uclm.es, 0000-0002-3662-7302 (P. Higueras)

***Corresponding author:** Samantha Jiménez-Oyola, sjimenez@espol.edu.ec

Table S1. Concentration of trace elements (mg/kg) in farmland soil samples

CODE	As	Cd	Cr	Cu	Ni	Pb	Zn	pH	Organic Matter (%)
S-01	37.8	1.06	99.5	151.37	66.72	8.69	150.52	7.18	2.46
S-02	38.64	1.07	101.04	152.75	67.58	8.77	154.22	7.32	4.88
S-03	369.54	1.09	92.38	215.59	62.77	12.86	220.6	7.06	2.77
S-04	339.94	1.2	93.24	212.19	63.99	14.35	207.4	7.24	3.13
S-05	333.74	1.19	91.58	211.59	62.44	14.54	204	6.61	6.09
S-06	421.74	1.28	83.7	207.39	59.17	15.16	216.6	6.27	5.42
S-07	23.54	1.6	77.24	100.91	50.21	14.14	180.68	6.28	1.00
S-08	23.86	1.94	84.16	114.33	51.41	13.08	173.58	7.58	4.58
EQG	12	2	65	63	50	60	200	6 - 8	-

EQG: Ecuadorian Quality Guideline for farmland soils (TULSMA 2015)

Table S2. Non-carcinogenic risk for adults and children

Crops	Heavy metal(loid)	HQ Children		HQ Adults	
		p50	p95	p50	p95
Corn	As	2.21E+01	3.81E+01	5.70E+00	7.76E+00
	Cd	1.24E+00	2.13E+00	3.22E-01	4.38E-01
	Cr	5.91E-02	1.01E-01	1.38E-02	1.87E-02
	Cu	6.58E-01	1.13E+00	1.70E-01	2.31E-01
	Ni	7.20E-01	1.23E+00	1.87E-01	2.54E-01
	Zn	4.62E-01	7.94E-01	1.20E-01	1.63E-01
Chives	As	2.49E+02	4.27E+02	6.46E+01	8.78E+01
	Cd	9.67E+01	1.66E+02	2.30E+01	3.13E+01
	Cr	9.17E-01	1.57E+00	2.84E-01	3.86E-01
	Cu	1.70E+00	2.91E+00	4.41E-01	5.99E-01
	Ni	9.14E-01	1.57E+00	2.37E-01	3.22E-01
	Zn	7.72E-01	1.32E+00	2.00E-01	2.72E-01
Green beans	As	2.95E+00	5.08E+00	7.67E-01	1.04E+00
	Cd	8.87E-01	1.52E+00	2.30E-01	3.13E-01
	Cr	1.48E-01	2.54E-01	3.83E-02	5.21E-02
	Cu	1.22E+00	2.10E+00	3.17E-01	4.32E-01
	Ni	3.19E-01	5.48E-01	8.29E-02	1.12E-01
	Zn	9.67E-01	1.66E+00	2.51E-01	3.41E-01
Celery	As	2.95E+00	5.08E+00	7.67E-01	1.04E+00
	Cd	6.74E+01	1.15E+02	1.65E+01	2.25E+01
	Cr	5.03E-01	8.63E-01	1.30E-01	1.77E-01
	Cu	1.75E+00	3.00E+00	4.54E-01	6.17E-01
	Ni	5.63E-01	9.68E-01	1.38E-01	1.87E-01
	Zn	2.19E+00	3.76E+00	5.68E-01	7.72E-01
Herbs	As	3.69E+01	6.35E+01	9.59E+00	1.30E+01
	Cd	4.88E+01	8.38E+01	1.26E+01	1.72E+01
	Cr	1.45E+00	2.49E+00	3.76E-01	5.11E-01
	Cu	3.52E+00	6.05E+00	9.14E-01	1.24E+00
	Ni	4.03E+00	6.93E+00	1.04E+00	1.42E+00
	Zn	2.01E+00	3.46E+00	5.23E-01	7.12E-01
Lettuce	As	3.69E+02	6.35E+02	9.59E+01	1.30E+02
	Cd	7.54E+01	1.29E+02	1.86E+01	2.53E+01
	Cr	9.46E+00	1.62E+01	2.33E+00	3.17E+00
	Cu	3.43E+00	5.89E+00	8.91E-01	1.21E+00
	Ni	1.87E+00	3.22E+00	4.87E-01	6.62E-01
	Zn	9.08E-01	1.56E+00	2.35E-01	3.20E-01
Turnips	As	7.48E+01	1.28E+02	1.95E+01	2.65E+01
	Cd	1.44E+02	2.48E+02	3.75E+01	5.10E+01
	Cr	1.48E-01	2.54E-01	3.83E-02	5.21E-02
	Cu	1.86E+00	3.20E+00	4.83E-01	6.56E-01
	Ni	1.12E+00	1.92E+00	2.93E-01	3.99E-01
	Zn	1.41E+00	2.43E+00	3.67E-01	5.00E-01

		HQ Children		HQ Adults	
		p50	p95	p50	p95
Cassava	As	2.95E+00	5.08E+00	5.37E-01	7.30E-01
	Cd	4.43E+00	7.62E+00	1.15E+00	1.56E+00
	Cr	2.95E-02	5.08E-02	7.67E-03	1.04E-02
	Cu	5.39E-01	9.26E-01	1.39E-01	1.90E-01
	Ni	9.67E-01	1.66E+00	2.51E-01	3.41E-01
	Zn	3.54E-01	6.09E-01	9.20E-02	1.25E-01
Carrots	As	5.74E+01	9.85E+01	1.48E+01	2.02E+01
	Cd	2.93E+01	5.03E+01	7.59E+00	1.03E+01
	Cr	8.87E-01	1.52E+00	2.30E-01	3.13E-01
	Cu	2.79E+00	4.80E+00	7.26E-01	9.88E-01
	Ni	8.16E-01	1.40E+00	2.11E-01	2.88E-01
	Zn	8.40E-01	1.44E+00	2.17E-01	2.96E-01

Table S3. Carcinogenic risk for adults and children.

**Crops**	**Heavy**	**CR Children**		**CR Adults**	
	**metal(loid)**	**p50**	**p95**	**p50**	**p95**
Corn	As	4.94E-04	1.00E-03	2.62E-04	1.39E-03
	Cr	1.11E-05	2.27E-05	5.90E-06	3.13E-05
Chives	As	5.63E-03	1.14E-02	2.98E-03	1.58E-02
	Cr	8.47E-05	1.72E-04	4.48E-05	2.38E-04
Green beans	As	6.68E-05	1.36E-04	3.54E-05	1.88E-04
	Cr	1.11E-05	2.27E-05	5.90E-06	3.13E-05
Celery	As	6.68E-05	1.36E-04	3.54E-05	1.88E-04
	Cr	3.78E-05	7.73E-05	2.00E-05	1.06E-04
Herbs	As	8.35E-04	1.70E-03	4.42E-04	2.35E-03
	Cr	1.09E-04	2.22E-04	5.78E-05	3.07E-04
Lettuce	As	8.35E-03	1.70E-02	4.42E-03	2.35E-02
	Cr	6.77E-04	1.38E-03	3.58E-04	1.90E-03
Turnips	As	1.70E-03	3.47E-03	9.03E-04	4.80E-03
	Cr	1.11E-05	2.27E-05	5.90E-06	3.13E-05
Cassava	As	6.68E-05	1.36E-04	3.54E-05	1.88E-04
	Cr	2.22E-06	4.54E-06	1.18E-06	6.27E-06
Carrots	As	1.29E-03	2.64E-03	6.87E-04	3.65E-03
	Cr	6.68E-05	1.36E-04	3.54E-05	1.88E-04

References

TULSMA. (2015). *Texto Unificado de Legislación Secundaria Medio Ambiental*. *Registro oficial órgano del gobierno del Ecuador*.
